# Metabolomic and transcriptomic analyses reveal grafting-regulated nutritional and flavor metabolites in tomato

**DOI:** 10.1186/s12870-026-09293-0

**Published:** 2026-06-22

**Authors:** Yuyuan Ma, Zhiyong Shao, Tonglin Wang, Zhixing Nie, Saisai Guo, Jirong Zheng

**Affiliations:** Vegetable Research Institute, Hangzhou Academy of Agricultural Sciences, Hangzhou, 310024 China

**Keywords:** Tomato, Grafting, Rootstock, Fruit quality, Transcriptome, Metabolome, Flavonoid biosynthesis

## Abstract

Grafting is a common agronomic strategy to improve growth, stress tolerance, and fruit quality in tomato. However, the molecular and metabolic mechanisms by which rootstocks regulate tomato fruit nutritional and flavor quality remain largely unclear. In this study, the tomato cultivar HZ504 was used as the scion and grafted onto three rootstocks (JZ, TLBM, JB), with self-grafted HZ504 as the control. Agronomic trait analysis showed that JZ rootstock significantly increased fruit number per plant and total soluble solids content. Integrated transcriptomic and metabolomic analyses revealed that JZ rootstock broadly altered the transcriptome and metabolome of scion fruits. Metabolites such as flavonoids, phenolic acids, terpenoids, and amino acids, all closely linked to flavor and nutrition, were elevated. On the transcriptome side, differentially expressed genes were mainly enriched in photosynthesis, starch and sucrose metabolism, and the phenylpropanoid/flavonoid biosynthesis pathways. At the pink ripening stage, JZ specifically upregulated key flavonoid pathway genes in a ripening-dependent manner, tightly correlating with increased flavonoid accumulation. Together, these results indicate that JZ rootstock enhances tomato yield and quality by reorganizing transcription and metabolic flux, particularly toward flavonoid biosynthesis. This work provides a practical basis for rootstock selection and sustainable production of high-quality tomatoes.

**Supplementary Information:**

The online version contains supplementary material available at https://doi.org/10.1186/s12870-026-09293-0.

## Introduction

Tomato (*Solanum lycopersicum* L.) is one of the most economically important vegetables globally, widely cultivated and consumed due to its rich nutritional value and unique flavor [1]. Fruit quality, which is comprehensively determined by flavor, nutritional composition, and antioxidant capacity, is closely dependent on the synthesis and accumulation of secondary metabolites [2]. These compounds constitute a large family of phytochemicals, including flavonoids, phenolic acids, terpenoids, alkaloids, and other specialized metabolites, all of which jointly shape the sensory and nutritional traits of ripe fruits [3–5]. As crucial bioactive constituents, they not only strengthen the nutritional quality and flavor characteristics of tomato fruits [6] but also enhance plant tolerance to various biotic and abiotic stresses [7]; notably, the health-promoting properties of tomato fruits are closely associated with the contents of amino acids, vitamin C, phenolics, anthocyanins, and flavonoid [8–10]. Collectively, tomato fruit flavor is determined by a combination of taste, volatile, and nutritional metabolites, each of which is shaped by multiple physiological and biochemical processes [11]. Therefore, enhancing the accumulation of beneficial secondary metabolites in tomato fruits has become a central objective in horticultural breeding and cultivation strategies, which is critical for satisfying consumer demands for high-quality produce and supporting the sustainable development of the tomato industry.

Grafting describes the technique whereby a scion is joined to a rooted rootstock, allowing subsequent healing and reconstruction of vascular tissues to generate a chimeric plant [12]. As an efficient and non-transgenic agronomic modification technique, it has been widely applied in horticulture and agriculture for crop improvement. In tomato production, grafting is extensively adopted worldwide due to its multiple advantages; its primary purpose is to prevent damage caused by soil-borne pathogenic bacteria in intensive production systems [13]. Beyond disease control, grafting can also enhance the water and nutrient utilization efficiency of tomato plants, increase yield, optimize fruit quality, and alleviate the adverse effects of abiotic stresses such as salinity, water deficit, extreme temperatures, and heavy metals [14]. In recent years, numerous studies have focused on the effects of grafting on tomato fruit quality; existing research has demonstrated that grafting can significantly alter the accumulation of various fruit metabolites, including soluble sugars, organic acids, flavonoids, and phenolic compounds, which are closely associated with fruit flavor and nutritional value. Using the cultivated tomato/Solanum pennellii heterografting system, Du et al. [15] identified 61 upwardly and 990 downwardly mobile mRNAs involved in RNA binding, photosynthesis, and heat response, demonstrating that long-distance mRNA movement contributes to stress adaptation in grafted tomato plants. At the interface of tomato scion and eggplant rootstock, histological observations revealed abnormally twisted vascular bundle arrangements, with transcriptomic analysis of scion tissues at the graft junction identifying significant expression changes in genes associated with lignin, xyloglucan metabolism and cell wall organization [16]. Beyond transcriptional regulation, recent studies have also uncovered the role of mobile small RNAs in grafting-induced phenotypic changes. Mobile 24 nt sRNAs derived from BABA-primed rootstocks were found to transmit long-lasting disease resistance to unprimed scions, with these sRNAs associating with genes showing primed expression during pathogen infection in fruit [17]. In eggplant, appropriate rootstocks have been shown to enhance fruit size, yield, and nutritional quality by increasing total soluble solids, phenolic compounds, sugars, and vitamin C [18]. In melon, grafting with compatible rootstocks improves fruit yield and weight, while also modulating the levels of flavonoids, phenolic compounds, amino acids, citrate, malate, and sucrose in the fruit pulp [19]. In plum, different rootstocks exert distinct effects on the accumulation of sugars, organic acids, and individual phenolic compounds including chlorogenic acid, neochlorogenic acid, cyanidin glycosides, and quercetin derivatives, which further influence fruit antioxidant capacity [20]. Even within melon landraces, rootstock genotype and scion–rootstock compatibility have been identified as key factors affecting sugar metabolism and the production of flavor-related metabolites [21]. These findings collectively highlight that the selection of suitable scion–rootstock combinations is critical for achieving favorable yield efficiency, plant vigor, and superior fruit quality. Moreover, the expression of desirable horticultural traits can vary markedly depending on the specific interaction between rootstock and scion genotype. Despite these consistent observations of graft-induced metabolic remodeling in multiple crops, the detailed molecular and regulatory mechanisms governing these changes remain largely underexplored.

Several recent studies have successfully applied multi-omics strategies to investigate the effects of grafting on tomato fruit quality. Wang et al. combined metabolomic and transcriptomic analyses to show that goji rootstock improves tomato quality through upregulation of flavonoid biosynthetic genes (*F3'H*, *F3'5'H*, *DFR*, *ANS*) and suppression of sucrose hydrolysis-related genes (*INV*, *SUS*, *BAM*) [22]. Similarly, Zhou et al. employed GC-MS and LC-MS to demonstrate that rootstock-scion interactions affect the accumulation of sugars, acids, and volatiles in grafted tomato fruits, and further identified mobile transcripts associated with these metabolic change [23]. Beyond metabolic profiling, Kodama et al. performed comprehensive transcriptomic, proteomic, and metabolomic analyses to assess the molecular effects of grafting onto transgenic rootstocks, demonstrating the high sensitivity of multi-omics approaches in detecting graft-induced variations [24]. Together, these studies highlight the effectiveness of integrated omics approaches in uncovering graft-mediated metabolic and transcriptional.

However, critical knowledge gaps persist. Grafting has been shown to positively impact plant growth and fruit quality, but the specific mechanisms by which it systematically modulates tomato fruit quality including flavor, nutritional composition and bioactive metabolite accumulation remain unclear. Most previous studies have focused on individual phenotypic or metabolic indicators rather than integrating comprehensive regulatory networks. Additionally, the coordinated regulatory mechanisms between gene expression and metabolite accumulation involved in grafting have not been fully explored, largely due to the lack of integrated transcriptomic and metabolomic analyses. The present study used grafted tomato plants as materials, systematically analyzed agronomic traits, fruit metabolome and gene expression patterns through transcriptomic and metabolomic approaches, with the aim of revealing the molecular mechanisms underlying graft-mediated quality improvement and providing a theoretical basis for the rational selection of rootstocks and the optimization of tomato cultivation techniques.

## Materials and methods

### Experimental materials and field experiment

The scion cultivar used in this experiment was tomato (*Solanum lycopersicum* L.) cv. HZ504. Three rootstock cultivars were selected for grafting, JZ (Jianzhuang No. 23), a commercial tomato rootstock from Mitsuo Company; TLBM (*Solanum torvum*, Tuolubamu), a wild eggplant rootstock with high resistance to soil-borne diseases [25]; and JB (Jinba 303), were selected for grafting a commercial tomato rootstock developed by Shenpihua Company. These three rootstocks represent different genetic backgrounds: JZ and JB are tomato (*Solanum lycopersicum*) rootstocks of distinct commercial origins, while TLBM is a different species (*Solanum torvum*), which is genetically distant from cultivated tomato. All plant materials, including the scion and rootstocks, were provided by Hangzhou Academy of Agricultural Sciences, Hangzhou, Zhejiang Province, China.

A self-grafted treatment of HZ504 was set as the control group to eliminate the potential impacts of grafting operation itself on plant growth and fruit quality. All seeds of scion and rootstock were subjected to surface disinfection with 75% ethanol for 30 s and 5% sodium hypochlorite for 10 min, followed by thorough rinsing with sterile distilled water, to ensure no seed-borne pathogens affected the experimental results. The disinfected seeds were germinated in a growth chamber at 25 ± 1℃ with a 16 h light/8 h dark photoperiod and a light intensity of 300 µmol·m⁻²·s⁻¹, and uniform seedlings with 2–3 true leaves were selected for grafting.

Grafting was performed using the splice (tube) grafting method as previously described [26, 27]. Briefly, rootstock seedlings were decapitated by a horizontal cut approximately 5 mm above the cotyledons using a sterile razor blade, and scion seedlings were cut at the corresponding position below the cotyledons. The scion was trimmed into a wedge shape (approximately 4–7 mm in length) and inserted into a vertical slit made at the cut surface of the rootstock. The graft union was secured with a flexible silicone grafting clip. Immediately after grafting, the grafted seedlings were placed in a healing chamber (25 ± 1 °C) under high relative humidity (≥ 95%) and shaded conditions to minimize transpiration and promote vascular reconnection. After 7 days, the humidity was gradually reduced and the plants were acclimatized to ambient greenhouse conditions before transplanting.

The experiment was conducted in the experimental field of Hangzhou Academy of Agricultural Sciences (Hangzhou, Zhejiang, China; 30°16′N, 120°12′E) from April to October. A randomized complete block design (RCBD) was adopted, with four treatments in total: HZ504/JZ (HZ504 scion grafted onto JZ rootstock), HZ504/TLBM (HZ504 scion grafted onto TLBM rootstock), HZ504/JB (HZ504 scion grafted onto JB rootstock), and the control group HZ504/HZ504 (self-grafted HZ504). Each treatment was replicated three times, with each replication consisting of an independent experimental plot. Each plot had an area of 4.8 m² (3 m × 1.6 m), with a row spacing of 0.6 m and a plant spacing of 0.4 m, allowing for 20 tomato plants per plot. To avoid cross-contamination between different treatments, a 1 m isolation distance was maintained between adjacent plots. Before transplanting, 3000 kg·ha⁻¹ of decomposed organic fertilizer and 750 kg·ha⁻¹ of compound fertilizer (N: P₂O₅:K₂O = 15:15:15) were applied as base fertilizer. During the growing period, conventional water and fertilizer management was implemented with drip irrigation to maintain soil relative water content at 70%–80% of field capacity. Pest and disease control followed integrated pest management (IPM) practices, including physical prevention and low-toxicity biological pesticides, with uniform application intervals across all treatments. When plants reached 30 cm in height, uniform vine hanging and pruning were performed, and all lateral branches were removed to maintain a single-stem growth pattern. All plots were managed under the same agronomic conditions throughout the growing period to ensure that the only variable was the rootstock type.

### Sample collection

Tomato fruits were collected from both the control and grafted treatments at four sequential ripening stages: mature green (MG), breaker (B), pink (P), and red ripened (R). Three biological replicates were set for each stage, with 3–5 fruits collected per replicate to ensure the representativeness of samples. All collected fruits were immediately frozen in liquid nitrogen and stored at -80℃ for subsequent metabolomic and transcriptomic analyses.

### Determination of agronomic traits

At the full fruit ripening stage, five representative plants were randomly selected from each plot for agronomic trait evaluation. The measured traits included plant height, leaf length, leaf width, stem diameter, fruit number per plant, single fruit weight, total soluble solids (TSS), and titratable acidity (acid).

Plant height was measured from the ground surface to the top of the main stem. Leaf length and width were determined using the 3rd fully expanded leaf from the apex of the main stem. Stem diameter was measured at 10 cm above the ground using a vernier caliper. Fruit number per plant was counted for all harvested fruits. Single fruit weight was calculated as the average weight of ten randomly selected ripe fruits per plot. TSS were determined using a digital refractometer with fruit juice extracted from ripe fruits. Acid was measured via titration with 0.1 M NaOH and expressed as citric acid equivalent [28]. All measurements were conducted in triplicate, and the mean values were used for subsequent statistical analysis.

### Plant metabolomics analysis

At the red ripened stage, tomato fruits were harvested, immediately frozen in liquid nitrogen, and stored at -80 °C until use.

For metabolite extraction, 10 g of fruit tissue was ground into a fine powder using a mortar and pestle with liquid nitrogen. Metabolites were extracted using 80% methanol (v/v) and incubated overnight at 4 °C with constant shaking. The resulting mixture was centrifuged at 12,000 rpm for 15 min at 4 °C, and the supernatant was filtered through a 0.22 μm polytetrafluoroethylene (PTFE) membrane prior to UPLC-MS/MS analysis.

Ultra-performance liquid chromatography-tandem mass spectrometry (UPLC-MS/MS) was performed on a Waters ACQUITY UPLC system (Waters Corporation, Milford, MA, USA) coupled to a Q Exactive Plus mass spectrometer (Thermo Fisher Scientific, Waltham, MA, USA). Chromatographic separation was achieved on an ACQUITY UPLC BEH C18 column (100 mm × 2.1 mm, 1.7 μm) at a column temperature of 40 °C with a flow rate of 0.3 mL·min⁻¹. The mobile phase consisted of solvent A (0.1% formic acid in water) and solvent B (0.1% formic acid in acetonitrile), with a linear gradient elution program.

Raw LC-MS data were preprocessed using Progenesis QI software (Waters, USA) to generate a three-dimensional data matrix (sample information, metabolite name, mass spectral response intensity). Internal standard peaks, noise, column bleed, and false positive peaks were removed, followed by deredundancy and peak integration. Metabolite identification was performed by matching the accurate mass-to-charge ratio (mass accuracy ≤ 5 ppm) and MS/MS fragment matching score ≥ 30 against the self-built plant-specific metabolite database (MJDBPM) of Majorbio Biotechnology Co., Ltd. (Shanghai, China), as well as public databases including the METLIN and KEGG databases. Principal component analysis (PCA) and partial least squares discriminant analysis (PLS-DA) were carried out using R software (v4.2.1). Differentially accumulated metabolites (DAMs) were identified with thresholds of variable importance in projection (VIP) ≥ 1 and *P* < 0.05 (t-test). Metabolic pathway enrichment analysis was performed based on the KEGG database using the clusterProfiler package in R, with *P* < 0.05 as the significance threshold. All metabolite identifiers, annotations, sample expression values, fold changes, VIP values, and *P*-values are provided in Supplementary Table S1.

### Transcriptomic analysis

Total RNA was extracted from MG stage fruit tissues of each treatment using a Plant Total RNA Extraction Kit (Tiangen Biotech, Beijing, China), following the manufacturer’s instructions. The purity and integrity of RNA were detected using a NanoDrop 2000 spectrophotometer (Thermo Fisher Scientific, USA) and an Agilent 2100 Bioanalyzer (Agilent Technologies, USA), respectively. Only RNA samples with an OD260/OD280 ratio of 1.8–2.0 and RNA integrity number (RIN) ≥ 8.0 were used for library construction. RNA sequencing (RNA-seq) was performed on an Illumina^®^ Stranded mRNA Prep, Ligation. Raw reads were filtered to remove low-quality reads (Q-value < 20), adapter sequences, and contaminating reads. The clean reads were mapped to the tomato reference genome (SL4.0) using Hisat2 software, and differentially expressed genes (DEGs) were identified using DESeq2 with |log2FC| ≥ 1 and adjusted *P*-value < 0.05 as the screening criteria. Gene functional annotation was performed using the GO and KEGG databases. GO and KEGG enrichment analyses were implemented using the clusterProfiler package in R, with *P* < 0.05 considered statistically significant. Procrustes analysis was conducted using the vegan package in R to evaluate the consistency between transcriptomic and metabolomic data. All gene identifiers, annotations, expression levels, fold changes, and adjusted *P*-values are listed in Supplementary Table S2.

### RNA extraction and quantitative real-time PCR (qRT-PCR) analysis

Total RNA was extracted from fruit samples fruit samples at the MG and P stages using a plant RNA extraction kit (Tiangen Biotech, Beijing, China). And 1 µg of high-quality RNA was used for first-strand cDNA synthesis with a PrimeScript RT reagent Kit with gDNA Eraser (Takara). Gene-specific primers were designed via Primer-BLAST (NCBI), and qPCR was performed on a Bio-Rad CFX96 system with SYBR^®^ Premix Ex Taq™ II [29]. Solyc11g005330 served as the internal reference, and relative gene expression was calculated using the 2^^(−ΔΔCt)^ method, with three biological and technical replicates [30].

### Statistical analysis

All statistical analyses were performed using R software (version 4.2.1). Differences in agronomic traits, fruit quality indexes, metabolite contents, and gene expression levels among treatments were tested by one-way analysis of variance (ANOVA). Means were compared using Tukey’s Honestly Significant Difference (HSD) test at the *P* < 0.05 significance level. All metabolomic and transcriptomic visualizations were generated using R (version 4.2.1).

## Results

### Differential effects of rootstock genotypes on tomato plant architecture and fruit quality attributes

**Fig. 1:**
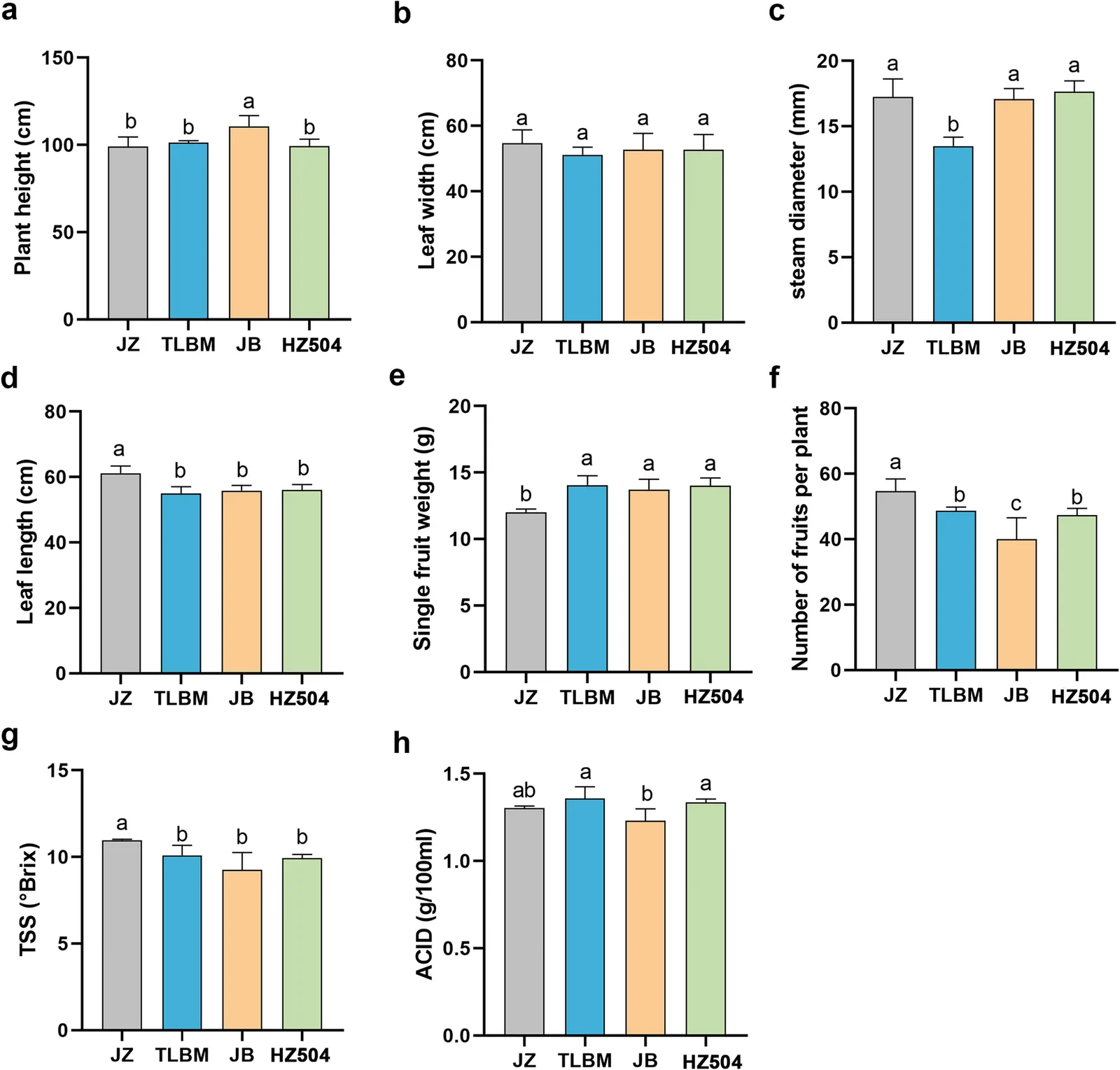
Effects of different rootstocks on agronomic traits and fruit quality of tomato scion HZ504. **a** Plant height; **b** Leaf width; **c** Stem diameter; **d** Leaf length; **e** Single fruit weight; **f** Number of fruits per plant; **g** Total soluble solids (TSS) content; **h** Titratable acid content. JZ, TLBM, JB: HZ504 grafted onto JZ, TLBM, JB rootstocks, respectively; HZ504: self-grafted HZ504. Data are presented as the mean ± standard error (SE). Different letters indicate significant differences (*P* < 0.05)

To investigate the comprehensive regulatory effects of rootstocks on tomato agronomic growth and fruit quality, this study used self-grafted HZ504 as the control and established three rootstock grafting combinations: JZ, TLBM, and JB. Plant height, stem and leaf traits are important vegetative growth indicators: plant height reflects overall growth vigor, stem diameter is closely linked to plant mechanical strength, lodging resistance and nutrient transport efficiency, while leaf morphological traits determine photosynthetic area and photosynthetic capacity [31]. All these vegetative characteristics collectively affect dry matter accumulation and further influence fruit yield and quality. The core quality and agronomic traits were measured and analyzed (Fig. 1). Plant height and leaf width remained comparable across all treatments, with values ranging from 99.00 cm to 110.67 cm and 51.17 cm to 54.70 cm, respectively (Fig. 1a, b). However, stem diameter was substantially affected by rootstock genotype (Fig. 1c). TLBM-grafted plants exhibited the smallest stem diameter (13.49 ± 0.68 mm), which was significantly reduced compared to HZ504 self-grafted controls (17.92 ± 0.77 mm), as well as JB-grafted plants (17.27 ± 0.87 mm). Leaf length differed significantly among graft combinations. JZ-grafted plants had the longest leaves (61.13 ± 2.19 cm), whereas TLBM (55.00 ± 2.00 cm), JB (55.83 ± 1.53 cm), and the control HZ504 (56.00 ± 1.53 cm) showed similar values that were approximately 8–10% shorter than those of JZ (Fig. 1d).

Regarding fruit-related traits, obvious differences existed between JZ and self-grafted HZ504 (Fig. 1e-h): HZ504 had a significantly higher single fruit weight (14.00 ± 0.59 g), while JZ-grafted plants had a higher TSS (10.85 ± 0.24%) and number of fruits per plant (54.67 ± 3.79). TLBM and JB-grafted plants had high single fruit weights similar to HZ504; JB had the lowest ACID content and number of fruits per plant (40.00 ± 6.56). TSS, a critical indicator of fruit sweetness composed of sugars, acids and small amounts of dissolved nutrients, showed the most notable difference between JZ and HZ504.

### Metabolic profiling reveals distinct differences between self-grafted and rootstock-grafted tomato fruits

**Fig. 2:**
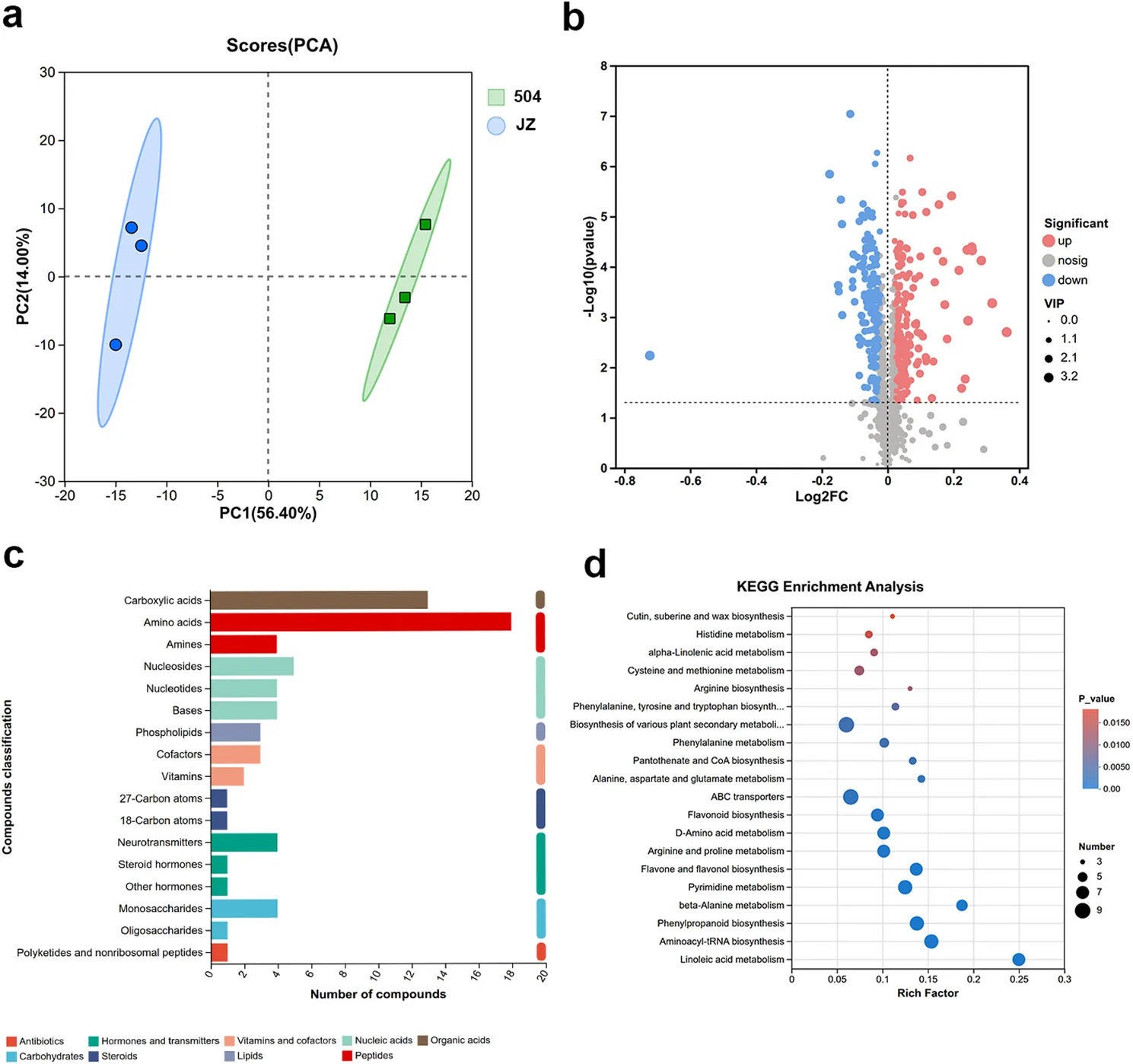
Metabolic profiling of JZ rootstock-grafted and self-grafted HZ504 tomato fruits. **a** PCA score plot showing the separation between self-grafted HZ504 (green) and JZ rootstock-grafted (blue) groups. **b** Volcano plot of differential metabolites between JZ rootstock-grafted and self-grafted HZ504 fruits. Red dots: upregulated metabolites; blue dots: downregulated metabolites; gray dots: no significant change. **c** Classification of differential metabolites by compound class. **d** KEGG enrichment bubble plot of differential metabolites. Rich factor (x-axis) indicates the proportion of differential metabolites in each pathway; point size represents the number of enriched metabolites; color gradient represents the *P*-value (red = higher significance)

Based on agronomic and fruit quality assessments, JZ-grafted fruits exhibited the highest TSS and a balanced titratable acid content, suggesting favorable taste potential. Therefore, JZ-grafted and self-grafted HZ504 fruits at the R stage were selected for widely targeted metabolomic analysis to explore the molecular basis underlying the improved organoleptic and nutritional quality. PCA was performed to assess the overall metabolic differences between the self-grafted control (HZ504) and the heterologous rootstock-grafted group (JZ). As shown in Fig. 2a, clear separation was observed between the two groups along the first principal component (PC1, 56.40%), while the second principal component (PC2, 14.00%) further distinguished intragroup variation, indicating good reproducibility within each group and significant metabolic divergence induced by different rootstocks. To confirm the separation and identify key discriminatory metabolites, PLS-DA was conducted (Figure S1). The PLS-DA score plot showed a robust separation between HZ504 and JZ groups, with Component 1 explaining 52.7% of the total variance and Component 2 explaining 15%, consistent with the PCA results and validating the distinct metabolic phenotypes triggered by heterologous rootstock grafting.

Differential metabolites between JZ and self-grafted HZ504 were identified using a volcano plot (Fig. 2b), with thresholds of |log₂(FC)| > 0, *P* < 0.05 and VIP > 1. In total, 374 differential metabolites were detected, of which 197 were upregulated and 177 were downregulated in the JZ rootstock-grafted group compared to the self-grafted HZ504 control, indicating a predominant trend of metabolic activation in fruits grafted onto the JZ rootstock. Classification of these differential metabolites (Fig. 2c) revealed that amino acids and peptides (18 metabolites) and carboxylic acids (13 metabolites) were the most abundant categories, followed by nucleosides, nucleotides, and monosaccharides. This distribution suggests that the JZ rootstock primarily modulates carbon and nitrogen metabolism, energy supply, and secondary metabolite biosynthesis in the HZ504 scion fruits. To further explore the biological functions of these differential metabolites, KEGG enrichment analysis was performed (Fig. 2d). The results showed significant enrichment in pathways related to amino acid metabolism, including histidine metabolism, cysteine and methionine metabolism, plant secondary metabolite biosynthesis, including flavonoid biosynthesis and phenylpropanoid biosynthesis, and lipid metabolism, including linoleic acid metabolism and cutin, suberine and wax biosynthesis. Notably, pathways involved in membrane transport (ABC transporters) and tRNA aminoacylation were also enriched, indicating that the JZ rootstock orchestrates both metabolic and transport processes in the scion to shape fruit quality and stress responses.

### Modulation of secondary metabolite profiles in tomato in response to grafting

**Fig. 3:**
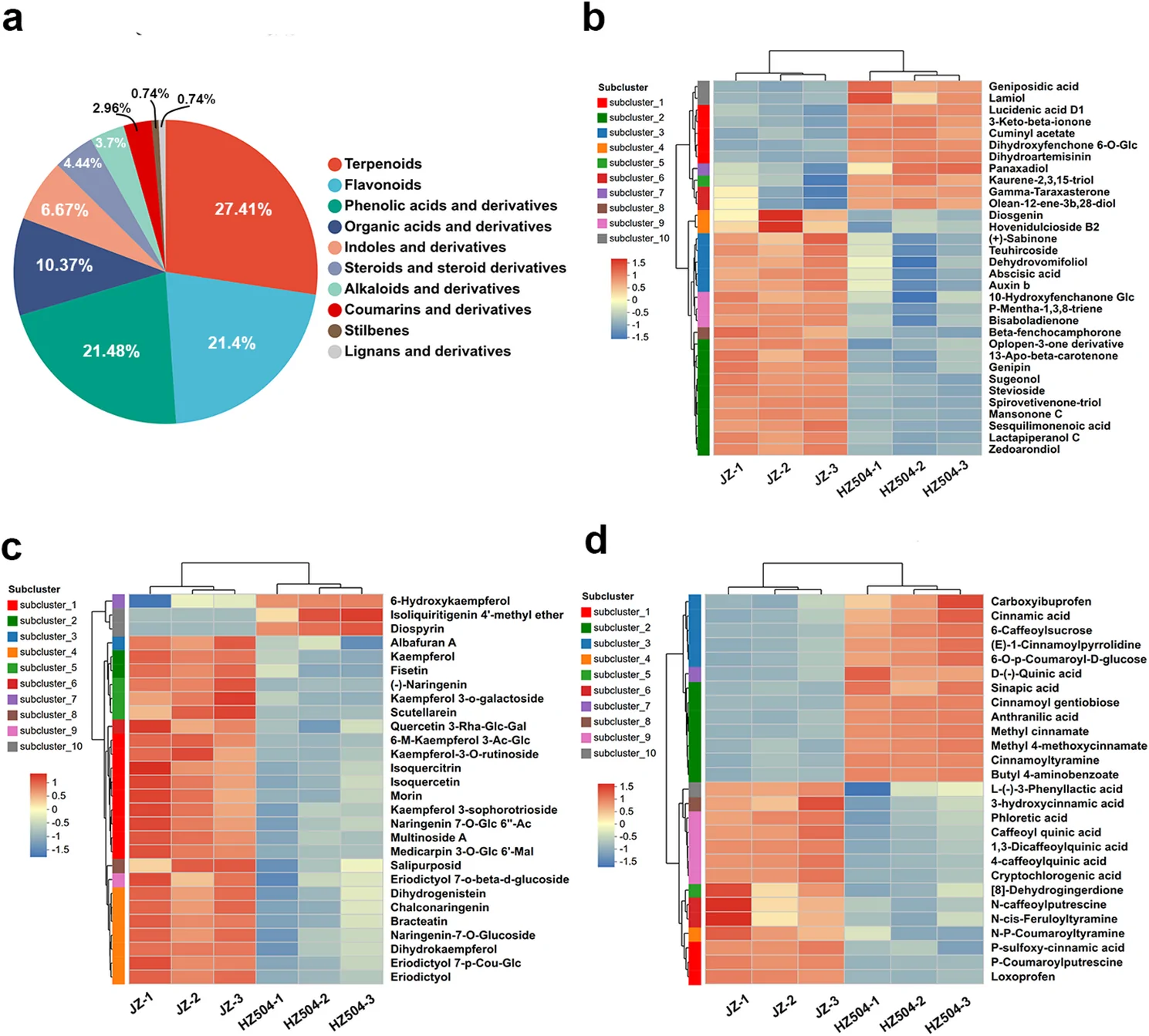
Grafting reshapes secondary metabolite profiles in tomato scions. **a** Pie chart showing the classification and relative proportion of all detected secondary metabolites. **b**–**d** Hierarchical clustering heatmaps of differentially accumulated metabolites in the categories of terpenoids (**b**), flavonoids (**c**), and phenolic acids and derivatives. **d** Samples are grouped as self-rooted (HZ504-1, HZ504-2, HZ504-3) and grafted (JZ-1, JZ-2, JZ-3). The color scale represents relative metabolite abundance (red = upregulated, blue = downregulated). Subclusters of metabolites with distinct accumulation patterns are highlighted by colored bars on the left

To investigate the impact of grafting on tomato secondary metabolism, widely targeted metabolomic analysis was performed on scion tissues of self-rooted (HZ504-1, HZ504-2, HZ504-3) and grafted (JZ-1, JZ-2, JZ-3) plants. A total of 135 secondary metabolites were identified and classified into major categories, including terpenoids, flavonoids, phenolic acids and derivatives, organic acids, alkaloids, and others (Fig. 3a). Terpenoids were the most abundant class, accounting for 27.41% of all detected metabolites, followed by flavonoids (21.40%) and phenolic acids and derivatives (21.48%).

A total of 32 terpenoid metabolites were identified as significantly different between HZ504 and JZ tomato plants, with detailed information listed in Table S2. Hierarchical clustering analysis revealed distinct terpenoid accumulation patterns between the two groups (Fig. 3b). Notably, multiple terpenoids associated with tomato aroma and flavor quality were significantly upregulated in JZ-grafted fruits. Dehydrovomifoliol, a crucial volatile compound contributing to tomato fruit aroma and a key catabolite of carotenoids, showed higher accumulation in JZ. Similarly, 13-apo-beta-carotenone and 3-keto-beta-ionone, which are derived from carotenoid degradation and closely related to fruit color formation and characteristic flavor, also exhibited significant changes. In addition, volatile monoterpenoids such as cuminyl acetate and p-mentha-1,3,8-triene, which contribute to the herbaceous and aromatic notes of tomato, were differentially accumulated between the two groups. Meanwhile, abscisic acid (ABA), a sesquiterpenoid hormone regulating fruit ripening and stress response, was more abundant in JZ. Other terpenoids including sesquiterpenoids, triterpenoids and monoterpenoid glycosides also displayed significant variations (Table S3).

Flavonoids, a major class of secondary metabolites crucial for tomato stress resistance, pigmentation and flavor formation (Fig. 3c). A large subcluster of flavonoids was markedly upregulated in JZ, while another subset was downregulated. Detailed annotation of these flavonoids (Table S4) revealed that the upregulated metabolites, which are closely associated with improved tomato fruit quality, primarily included flavanones, flavonols, and their glycoside derivatives, all of which play vital roles in enhancing tomato appearance, taste and nutritional value. Among the quality-beneficial flavonoids, eriodictyol and its glycoside derivative eriodictyol 7-(6-trans-p-coumaroylglucoside) showed the most significant upregulation. Naringenin and its glycoside derivatives, key contributors to tomato fruit flavor and antioxidant properties, were also significantly upregulated in grafted plants: (-)-naringenin, naringenin 7-O-beta-D-glucoside 6’’-acetate, and naringenin-7-O-glucoside. Most flavonols and their glycosides related to fruit pigmentation and nutrition were upregulated in JZ, such as kaempferol and its derivatives, quercetin 3-rhamnosyl-(1->6)-glucosyl-(1->6)-galactoside and morin, which promote fruit color formation and enrich nutritional composition. Only a few quality-related flavonoids were mildly downregulated, such as 6-hydroxykaempfero.

Phenolic acids and their derivatives also displayed distinct accumulation patterns (Fig. 3d), which is consistent with their crucial roles in tomato physiology, stress adaptation and fruit quality formation. JZ showed significantly increased levels of caffeic acid, sinapic acid, and p-coumaric acid—core intermediates in the phenylpropanoid pathway that serve as pivotal precursors for the biosynthesis of more complex phenolic compounds, including the quality-beneficial quinic acid derivatives and cinnamic acid derivatives identified in our study (Table S5).

### Transcriptomic profiling across graft combinations in tomato

Transcriptome sequencing was performed on three biological replicates each for the JZ rootstock-grafted and self-grafted HZ504 tomato groups, to characterize the molecular changes underlying graft-induced fruit quality variation. Data quality control (Table S6) showed that all samples generated 6.34–6.56 Gb raw bases, with 98.89–99.22% retained as clean reads after filtering. The error rate was 0.0116–0.0117%, Q20 and Q30 values were 99.27–99.32% and 96.36–96.54%, respectively, and GC content ranged from 42.68 to 42.91%, confirming high-quality data suitable for downstream analysis.

**Fig. 4:**
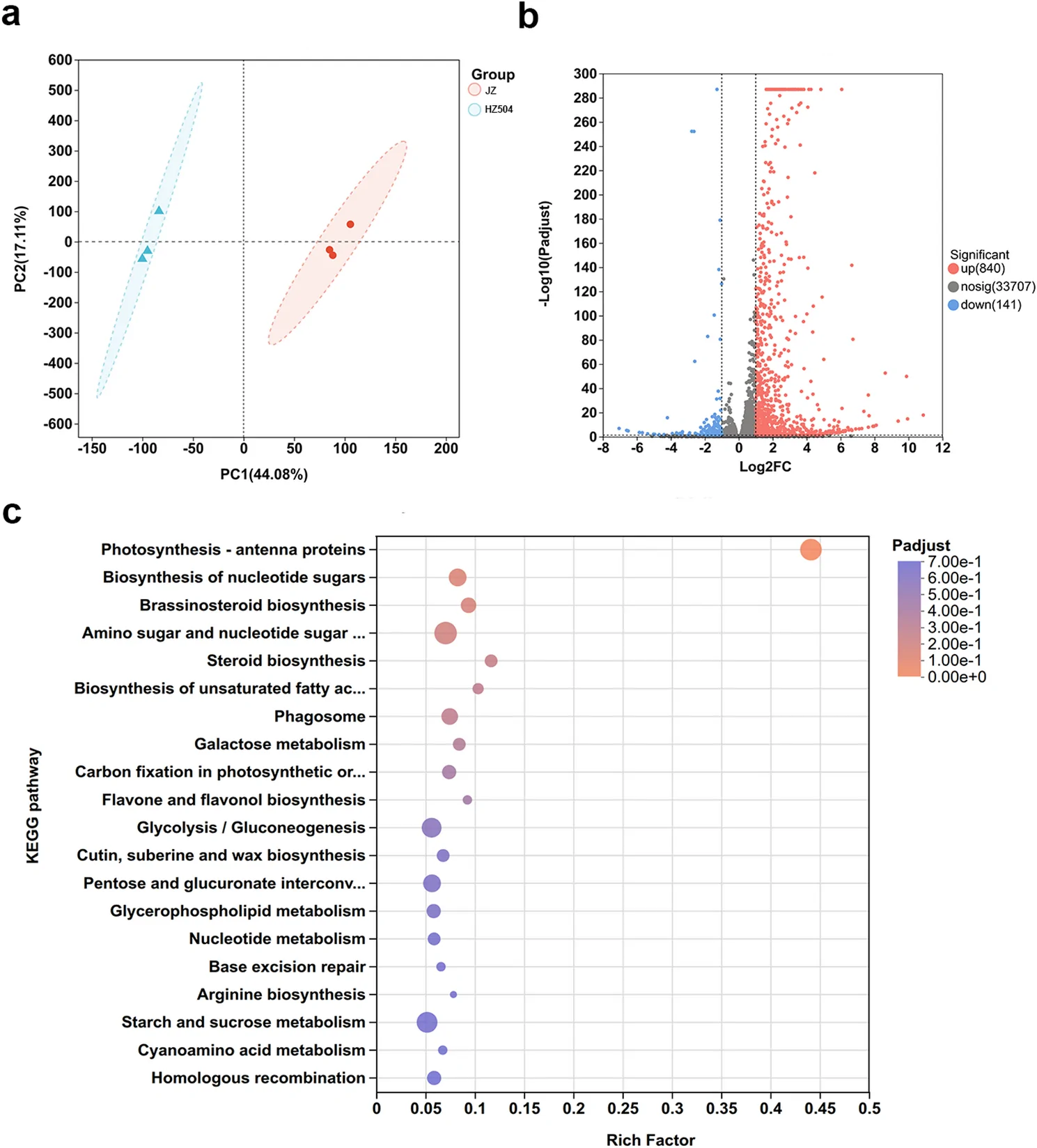
Transcriptomic profiling of JZ rootstock-grafted and self-grafted HZ504. **a** PCA score plot of gene expression profiles in JZ rootstock-grafted and self-grafted HZ504 groups. Variance explained by each component is shown in parentheses. **b** Volcano plot of DEGs. X-axis: log₂ fold change (Log₂FC); Y-axis: −log₁₀(Padjust). Red/blue/gray dots represent upregulated, downregulated, and non-significant genes, respectively. **c** KEGG enrichment bubble plot of top pathways. X-axis: Rich Factor; bubble color indicates Padjust value; bubble size represents the number of annotated genes

PCA revealed that PC1 (44.08%) and PC2 (17.12%) explained 61.20% of total variation, with clear separation between JZ and HZ504 groups and tight clustering of biological replicates, indicating distinct expression patterns and high experimental reproducibility. Using thresholds of |Log₂FC| ≥ 1 and Padjust < 0.05, a total of 981 DEGs were identified, including 840 upregulated and 141 downregulated genes (Fig. 4b). The predominance of upregulated genes suggests that grafting primarily activates gene expression related to fruit quality formation, and to further elucidate the biological functions of these DEGs and their potential roles in regulating fruit quality, KEGG pathway enrichment analysis was performed. KEGG pathway enrichment analysis (Fig. 4c) highlighted significant enrichment of pathways including Photosynthesis-antenna proteins, Flavone and flavonol biosynthesis, Glycolysis/Gluconeogenesis, Starch and sucrose metabolism, and Biosynthesis of unsaturated fatty acids. These pathways are closely linked to carbon allocation, antioxidant compound accumulation, and lipid metabolism, aligning with the observed changes in fruit quality-related metabolite. The enrichment of these genes reflects the potential transcriptional regulation of carbon metabolism and secondary metabolite biosynthesis pathways in response to grafting with the JZ rootstock.

To further characterize the functional annotations of the identified DEGs, GO enrichment analysis was performed alongside KEGG pathway analysis. Enriched GO terms were categorized into biological processes (BPs), cellular components (CCs), and molecular functions (MFs). In biological processes, the top enriched terms included photosynthesis, light harvesting, photosystem I/II assembly, and cell wall organization or biogenesis. For cellular components, DEGs were predominantly enriched in the photosystem, organelle outer membrane, and external encapsulating structure. In molecular functions, the most prominent enriched terms were chlorophyll binding, tetrapyrrole binding, and catalytic activities related to carbohydrate and polysaccharide metabolic processes (Figure S2).

### Transcriptomic and metabolomic integrative analysis

**Fig. 5:**
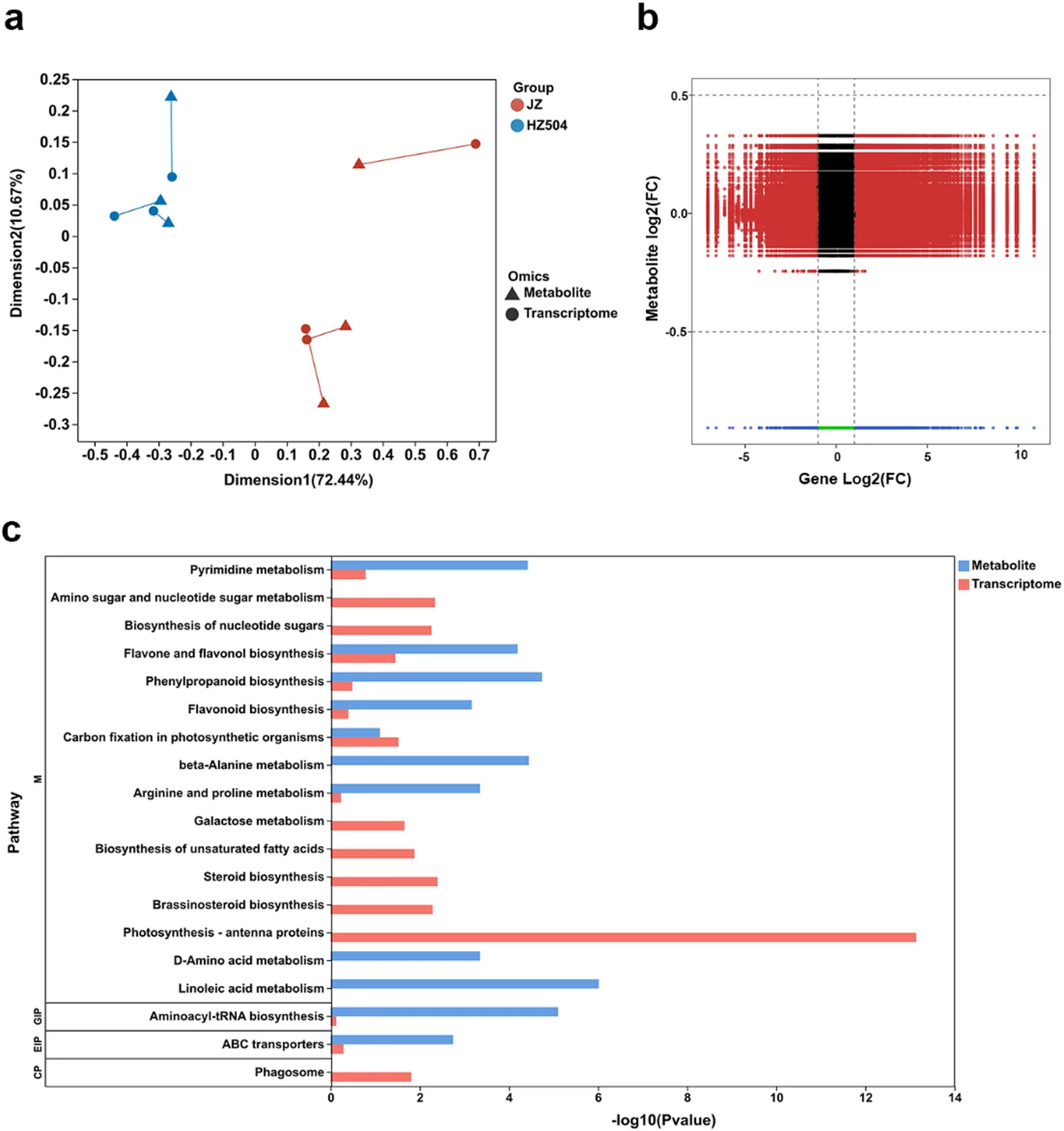
Integrated transcriptomic and metabolomic analysis of tomato grafted with rootstock JZ vs. self-grafted HZ504. **a** Procrustes analysis showing the synergistic variation between transcriptome and metabolome. Circles and triangles represent transcriptomic and metabolomic data, respectively; red and blue indicate the JZ-grafted group and self-grafted HZ504 control group, respectively. M² = 0.267, *p* = 0.02. **b** Nine-quadrant correlation plot of Log₂ (fold change) values for DEGs and DAMs, based on Pearson correlation coefficients > 0.8. X-axis: Gene Log₂(FC); Y-axis: Metabolite Log₂(FC). Different colors and quadrants represent distinct regulatory patterns and interactions. **c** Joint KEGG enrichment analysis of transcriptome and metabolome. Blue bars represent metabolite enrichment, red bars represent transcript enrichment; the x-axis shows -log₁₀(*P*-value), indicating the significance of pathway enrichment

To systematically decipher the molecular mechanisms underlying the regulatory effects of grafting with rootstock JZ on tomato growth and quality, we performed integrative analysis of transcriptomic and metabolomic data, using self-grafted HZ504 as the control. Procrustes analysis showed a significant concordance between the transcriptome and metabolome (M² = 0.267, *p* = 0.02), confirming that the two omics profiles displayed strong synergistic changes in response to rootstock grafting (Fig. 5a). Samples of the self-grafted control and JZ rootstock-grafted group were clearly separated along Dimension 1, which accounted for 72.44% of the total variation, indicating that grafting with JZ rootstock caused dramatic and distinctive molecular alterations in tomato. Transcriptomic and metabolomic data points from the same biological replicate were closely clustered within each group, especially in self-grafted HZ504, verifying the high repeatability and reliability of the multi-omics data.

The nine-quadrant correlation plot of Log₂ (fold change) values further illustrated the regulatory relationship between genes and metabolites (Fig. 5b). Most gene-metabolite pairs were distributed in the red region, where most differentially expressed genes were significantly upregulated, while differential metabolites showed relatively mild changes. These results indicated that the transcriptome responded more rapidly to grafting signals and initiated primary regulatory events, whereas the metabolome underwent relatively delayed and steady adjustments.

In accordance with the global correlation results, joint KEGG enrichment analysis further revealed the key biological pathways co-regulated or specifically regulated at the transcriptomic and metabolomic levels (Fig. 5c). At the transcript level, the Photosynthesis - antenna proteins pathway was extremely significantly enriched. This result was highly consistent with the agronomic traits, in which improved photosynthetic efficiency contributed to the enhanced growth vigor of grafted tomato plants. At the metabolome level, pathways including Linoleic acid metabolism, Pyrimidine metabolism, and Arginine and proline metabolism were significantly enriche. Notably, Phenylpropanoid biosynthesis and its downstream Flavonoid biosynthesis and Flavone and flavonol biosynthesis pathways were co-enriched in both transcriptome and metabolome, forming the core synergistic regulatory module in this study. The co-enrichment indicated that JZ rootstock not only mediated the expression of key structural genes in phenylpropanoid and flavonoid biosynthesis, but also promoted the accumulation of representative secondary metabolites associated with fruit antioxidant activity and nutritional quality.

### Flavonoid biosynthesis and gene–metabolite networks in grafted tomatoes

Flavonoids, particularly flavonol glycosides, are pivotal bioactive components that contribute to the nutritional quality, antioxidant capacity, and shelf life of tomato fruits. Accordingly, we performed an integrated transcriptomic and metabolomic analysis to dissect the coordinated regulation of the flavonoid biosynthetic pathway in fruits from the JZ-grafted group and the self-grafted HZ504 control group.

**Fig. 6:**
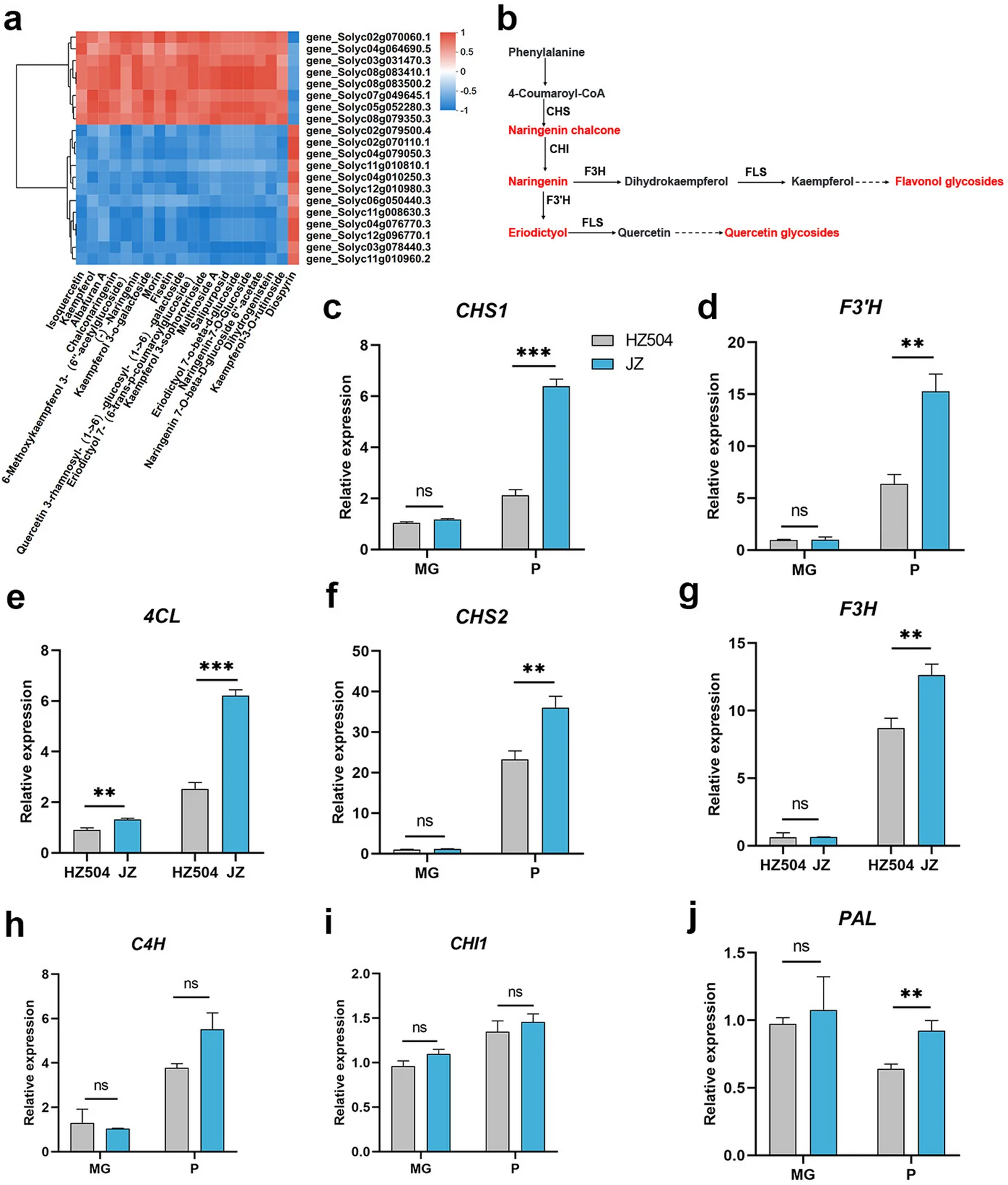
Transcriptomic and metabolomic analysis of the flavonoid biosynthetic pathway in JZ-grafted tomato fruits. **a** Pearson correlation heatmap of flavonoid pathway-related genes and metabolites. The color scale represents the correlation coefficient (red, positive correlation; blue, negative correlation). **b** Schematic diagram of the canonical flavonoid biosynthetic pathway. Red text indicates metabolites significantly accumulated in JZ-grafted fruits compared to the self-grafted HZ504 control. **c**–**j** qRT-PCR analysis of key structural genes in the flavonoid pathway at the MG and P stages. Data are presented as the mean ± standard deviation (SD). Significant differences between JZ and HZ504 at each stage are indicated by asterisks (** *P* < 0.01; *** *P* < 0.001); ns, not significant

To explore the regulatory relationships between flavonoid biosynthetic genes and metabolites, we constructed a Pearson correlation heatmap using 16 core differentially expressed genes (DEGs) and 18 key differentially accumulated metabolites (DEMs) associated with the flavonoid pathway (Fig. 6a). The heatmap revealed two distinct, well-separated regulatory modules. The upregulated module was enriched in key structural genes of the flavonoid biosynthetic pathway, including *Solyc03g031470*, *Solyc04g079050*, *Solyc08g079350*, and *Solyc08g083410*. These genes were significantly upregulated in JZ fruits and exhibited strong positive correlations with the majority of flavonoid metabolites that were highly accumulated in grafted plants, such as naringenin, eriodictyol, kaempferol glycosides, and quercetin glycosides. In contrast, the downregulated module primarily comprised peroxidases, acyltransferases, and other accessory enzymes, which were significantly downregulated in JZ and showed strong negative correlations with the abovementioned functional flavonoids.

To further characterize the complex regulatory relationships within the flavonoid pathway, we constructed a gene-metabolite association network (Figure S3). In this network, red nodes represent DEGs, blue nodes represent DEMs, green lines indicate negative correlations, and red lines indicate positive correlations. The network identified several hub genes, including *Solyc04g076770* and *Solyc02g083480*, which displayed the highest connectivity and were significantly associated with multiple flavonoid metabolites, confirming their central roles in the regulatory network. The overall network topology was consistent with the heatmap results, showing that core structural genes were predominantly positively correlated with functional flavonoid metabolites, while accessory genes were negatively correlated.

Based on the correlation analysis, we mapped the expression patterns of key genes onto the canonical flavonoid biosynthetic pathway to elucidate the regulatory network triggered by grafting (Fig. 6b). The schematic illustrates the stepwise conversion of phenylalanine into various flavonoid intermediates and end products, highlighting the enzymatic reactions catalyzed by key structural enzymes including PAL, 4CL, CHS, CHI, F3H, F3’H, and FLS. Notably, several core intermediates, including naringenin, eriodictyol, and their downstream derivatives, are marked in red, indicating their significantly higher accumulation in fruits grafted onto the JZ rootstock compared with the self-grafted HZ504 control.

To validate the transcriptomic profiling and further explore the ripening-associated dynamics of flavonoid biosynthesis, we conducted qRT-PCR analysis on key structural genes of the flavonoid pathway at both the MG and P stages (Fig. 6c–j). These genes, including *PAL*, *4CL*, *C4H*, *CHS1*, *CHS2*, *CHI1*, *F3H*, and *F3’H*, have been well characterized as core components responsible for flavonoid accumulation in tomato fruits [32]. Transcriptomic analysis, performed at the MG stage, identified the flavonoid biosynthetic pathway as a major target of JZ rootstock-mediated regulation, despite the fact that many core enzymatic genes within this pathway did not exhibit significant differential expression in the initial transcriptome data. However, qRT-PCR analysis revealed a clear stage-specific pattern in gene expression: at the MG stage, no significant differences were observed for most tested genes between JZ-grafted and self-grafted HZ504 fruits. In contrast, at the P stage, the expression levels of core structural genes including *CHS1*, *CHS2*, *F3H*, *F3’H*, *4CL*, and *PAL* were significantly upregulated in JZ-grafted fruits compared to the control. Notably, the expression of *C4H* and *CHI1* remained unchanged at both developmental stages. These findings suggest that the JZ rootstock triggers a delayed but robust transcriptional activation of the flavonoid pathway during fruit ripening, which is tightly coordinated with the metabolic accumulation of flavonoid metabolites.

## Discussion

Grafting is an effective agronomic measure to improve crop stress resistance and fruit quality, and it plays a crucial role in the sustainable development of the vegetables industry [33, 34]. Through exploring the interaction between different rootstocks and scion, our study revealed that rootstock types exert a pivotal influence on tomato plant growth and the metabolic composition of fruits. JZ standing out by increasing both fruit number per plant and total soluble solids (TSS). JB, in contrast, promoted greater plant height but without a corresponding yield or quality benefit. This divergence suggests that rootstock-mediated effects on vegetative growth and reproductive output are not necessarily coupled, a point often overlooked in grafting studies that focus solely on yield. JB increased plant height but not fruit number or TSS, implying that gibberellin or auxin signaling may favor stem elongation at the expense of carbon allocation to fruits.

Rootstock–scion interaction is known to reshape primary and secondary metabolism in fruit crops, including watermelon, grapevine, and cucumber [33, 35, 36]. In the present study, widely targeted metabolomic analysis revealed that grafting with JZ rootstock led to substantial metabolic changes in red-ripened tomato fruits. A total of 185 differential metabolites were detected, with a pronounced trend toward upregulation, indicating that suitable rootstocks predominantly activate metabolic pathways related to nutritional quality. These metabolites were mainly enriched in amino acid metabolism, plant secondary metabolite biosynthesis, ABC transporters, and lipid metabolism (Fig. 2c). Notably, the preferential activation of phenylpropanoid and flavonoid pathways suggests a strategic metabolic shift toward antioxidant and functional metabolite accumulation, rather than general metabolic stimulation. This pattern reveals that JZ rootstock may optimize resource partitioning by prioritizing secondary metabolism that supports both fruit quality and stress resilience. Such coordinated metabolic rearrangement provides a mechanistic explanation for the increased total soluble solids and improved nutritional composition observed in phenotypic assays. This regulatory mode is consistent with observations in grafted eggplant, where compatible rootstocks enhanced taste and functional value by modulating sugar, organic acid, and phenolic metabolism [18].

Secondary metabolites, including terpenoids, flavonoids, and phenolic acids, are key determinants of tomato nutritional value, flavor, and antioxidant capacity [37, 38]. In this study, a total of 374 metabolites were detected, among which terpenoids, flavonoids, and phenolic acids were the most abundant. Further analysis showed that multiple terpenoids related to aroma and ripening regulation were differentially accumulated in JZ-grafted fruits, including dehydrovomifoliol, 13-apo-beta-carotenone, and abscisic acid. Dehydrovomifoliol and 13-apo-beta-carotenone are important catabolites of carotenoids and contribute to tomato characteristic aroma and flavor quality [39, 40]; their increased accumulation suggests that JZ rootstock improves fruit flavor via modulating terpenoid metabolism. ABA, as a key sesquiterpenoid hormone, can promote fruit ripening and stress tolerance [41, 42], and its higher level in JZ-grafted fruits indicates enhanced ripening regulation and adaptability (Table S3). Meanwhile, flavonoids and phenolic acids were extensively upregulated in JZ-grafted fruits, such as naringenin, eriodictyol, kaempferol derivatives, caffeic acid, sinapic acid, and p-coumaric acid (Fig. 3). Naringenin and eriodictyol are core intermediates of flavonoid biosynthesis and contribute to fruit taste and antioxidant capacity; Kaempferol and its glycoside derivatives enhance nutritional value and appearance quality [43]. The increased abundance of caffeic acid, sinapic acid, and p-coumaric acid indicates enhanced carbon flow into the phenylpropanoid pathway, which provides sufficient precursors for downstream functional metabolites [44]. Collectively, these changes reflect that JZ rootstock enhances the nutritional and functional quality of tomato fruits by promoting the biosynthesis and accumulation of flavonoids and phenolic acids.

The distinct metabolic and transcriptomic changes induced by the JZ rootstock imply that rootstock-derived long‐distance signals, such as hormones, mobile RNAs, peptides, or proteins, may transmit regulatory information from rootstock to scion and modulate transcription factors or rate‐limiting enzymes in the scion fruit [45, 46]. In grafted horticultural crops, such root-to-shoot signals are increasingly recognized as critical regulators that integrate underground nutrient availability, hormone balance, and stress status into aboveground growth and fruit quality formation. Here, the extensive upregulation of photosynthesis-related genes points to a possible enhancement of carbon fixation efficiency by JZ rootstock-derived signals. An elevated carbon supply would not only support increased fruit number but also provide carbon skeletons and energy for the biosynthesis of soluble sugars, amino acids, and downstream secondary metabolites, including flavonoids, phenolic acids, and terpenoids. Thus, JZ rootstock may reconfigure source–sink relationships to prioritize carbon allocation toward fruit nutritional and flavor quality.

Several aspects of this hypothesis warrant further investigation. First, the identity of the mobile signals remains unknown. Previous studies have documented the movement of hormones (ABA, auxin, strigolactones), mRNAs, and small RNAs across graft junctions in tomato and other species. Whether any of these candidate molecules underlie the observed effects is an open question that future sap profiling or comparative transcriptomics of the graft union could address. Second, increased transcription of photosynthetic genes does not directly equate to higher carbon assimilation; actual photosynthetic capacity must be verified using gas exchange or chlorophyll fluorescence measurements [47]. Third, even if carbon fixation is enhanced, how this translates into higher fruit TSS and fruit number depends on source–sink dynamics. Increased precursor supply may be partitioned differently depending on sink strength, phloem loading capacity, and fruit developmental programs [48]. The higher fruit number in JZ-grafted plants implies altered sink activity, which may further feedback-regulate source strength. Disentangling these source–sink interactions in the context of rootstock-mediated signaling is a valuable direction for future research. Finally, while carbon precursors are necessary for secondary metabolite biosynthesis, the transcriptional activation of flavonoid pathway genes observed at the pink stage suggests that direct regulatory control (MYB transcription factors) likely plays a major role, potentially acting in concert with increased substrate availability. Thus, while long-distance signaling provides a sound framework for understanding graft-induced quality improvement, the complete molecular cascade from rootstock to fruit metabolism remains to be established. Future studies combining mobile RNA sequencing, hormone quantification, and physiological analysis of carbon partitioning will help clarify the precise mechanisms underlying rootstock–scion crosstalk.

Flavonoids are synthesized via the well-characterized phenylpropanoid pathway, in which PAL, 4CL, CHS, CHI, F3H, F3′H, and FLS act as rate-limiting enzymes [49]. In our study, the majority of these core genes were significantly upregulated in JZ-grafted fruits at the P stage, and their expression levels showed strong positive correlations with the accumulation of naringenin, eriodictyol, and their glycosylated derivatives. This suggests that JZ rootstock exerts a “push” effect on the entire flavonoid biosynthetic flux. More intriguingly, we observed a “pull” effect from the downregulated module: peroxidases and acyltransferases (Fig. 6a), which are known to catalyze oxidative polymerization or conjugation of flavonoids [50, 51], were significantly downregulated in JZ fruits. Peroxidases can reduce the pool of soluble flavonoids by forming insoluble complexes [50], while acyltransferases often modify flavonoid stability and compartmentation [51]. Therefore, the downregulation of these accessory enzymes likely minimizes flavonoid turnover or degradation, thereby further elevating net accumulation. A similar mechanism has been reported in ripening tomato fruits, where decreased peroxidase activity coincides with anthocyanin retention [52]. Our gene-metabolite association network identified *Solyc04g076770* and *Solyc02g083480* as hub genes with the highest connectivity. The functions of these genes remain uncharacterized, but their strong negative correlations with flavonoid metabolites suggest that they might encode negative regulators or degradation enzymes. Future functional studies are needed to validate their roles.

One of the most striking findings from our qRT‑PCR analysis is the stage‑specific expression pattern. At the MG stage, none of the tested core genes differed significantly between JZ‑grafted and self‑grafted controls. However, at the P stage, *PAL*, *4CL*, *CHS1*, *CHS2*, *F3H*, and *F3'H* were all markedly upregulated in JZ fruits. This indicates that the JZ rootstock does not constitutively activate flavonoid genes; instead, it triggers a delayed but potent transcriptional activation that coincides with fruit ripening. Multiple complementary mechanisms may account for this stage-dependent regulatory pattern. The ripening-specific activation of flavonoid pathway genes is likely governed by developmental context dependency, whereby rootstock-derived long-distance signals cannot exert their regulatory functions until ripening-associated regulatory factors (RIN, NOR, and CNR) accumulate sufficiently in fruit tissues [53]. Alternatively, the rootstock might primarily modulate the ripening program itself, and the upregulation of flavonoid genes is an indirect consequence of accelerated or intensified ripening. It is also plausible that systemic grafting signals require a certain accumulation period to reach biologically effective concentrations in fruit flesh, consistent with previous studies reporting that grafting-induced metabolic alterations tend to be more prominent at late developmental stages [54]. Notably, *C4H* and *CHI1* expression remained unchanged at both stages, indicating that not all structural genes are equally responsive to rootstock signals, possibly due to differences in promoter architecture or upstream regulators.

Although our integrated transcriptomic and metabolomic analyses provide new insights into rootstock-mediated regulation of tomato fruit quality, this study has several limitations that need to be addressed in future work. In the present study, transcriptomic analysis was performed at the mature green stage, while metabolomic detection was focused on the red ripened stage, which limited our ability to directly associate dynamic gene expression with real-time metabolite accumulation at the same developmental phase. In addition, the long-distance signaling events and upstream regulatory mechanisms that mediate rootstock–scion communication, such as mobile RNAs, hormonal signals, transcription factors, or epigenetic modifications, remain to be further explored. Future studies adopting parallel multi-omics analysis at consistent fruit developmental stages will help establish a more continuous and precise regulatory network. Furthermore, integrating epigenomic profiling, proteomics, and functional gene validation will contribute to clarifying the molecular basis of graft-induced metabolic changes and facilitate the development of molecular markers for rational rootstock selection in tomato quality breeding.

## Conclusions

This study establishes that the choice of rootstock serves as a potent determinant of fruit compositional quality in tomato. When the HZ504 scion was combined with the JZ genotype, fruits exhibited a substantial rise in soluble solids, marking this particular graft union as advantageous for flavor related traits. Metabolite profiling of the resulting fruits uncovered a broad scale shift in secondary chemistry, with prominent gains observed among flavonoid glycosides, phenylpropanoid intermediates, and certain volatile terpenoids, classes intimately linked to taste perception and dietary value. Parallel analysis of gene expression patterns indicated that these chemical alterations stem from a restructured transcriptional landscape within the fruit, wherein pathways governing light capture, carbon partitioning, and phenylalanine flux underwent significant adjustment. The enzymatic machinery of flavonoid production was selectively engaged during the pink stage of ripening under JZ influence, coinciding with the suppression of transcripts putatively involved in metabolite oxidation or conjugation. These coordinated changes led to greater accumulation of phenolic antioxidants in grafted fruits compared with self-grafted controls. Collectively, our multi-omics findings highlight that optimized rootstock–scion pairing remodels primary and secondary metabolic networks via stage-specific transcriptional regulation, offering a feasible non-transgenic strategy to improve tomato fruit nutritional and flavor quality in practical cultivation.

## Supplementary Information

Supplementary Material 1.

Supplementary Material 2.

## Data Availability

All original data supporting the findings of this study are provided within the article and the supplementary materials. Any additional data or materials not included in the manuscript can be obtained from the corresponding author upon reasonable request.

## Abbreviations

RCBD: randomized complete block design
MG: mature green
B: breaker
P: pink
R: red ripened
TSS: total soluble solids
Acid: titratable acidity
UPLC-MS/MS: Ultra-performance liquid chromatography-tandem mass spectrometry
PLS-DA: partial least squares discriminant analysis
RNA-seq: RNA sequencing
DEGs: differentially expressed genes
qRT-PCR: quantitative real-time PCR
BPs: biological processes
CCs: cellular components
MFs: molecular functions
DEMs: differentially accumulated metabolites
CHS: chalcone synthase
F3’H: flavonoid 3’-hydroxylase
4CL: 4-coumarate CoA ligase
F3H: naringenin 3-dioxygenase
C4H: Cinnamate-4-hydroxylase
CHI: chalcone isomerase
PAL: Phe ammonia lyase
