# Supplementary material for: Metabolomic and transcriptomic analyses reveal grafting-regulated nutritional and flavor metabolites in tomato
Source: BMC Plant Biol. 2026 Jun 22;26:1522. doi: 10.1186/s12870-026-09293-0 (PMC13543641; doi:10.1186/s12870-026-09293-0)
Supplement: Supplementary file 1 [file 12870_2026_9293_MOESM1_ESM.docx]

**Figure S1**. PLS-DA score plot of metabolic profiles in HZ504 self-grafted and JZ rootstock-grafted tomato fruits.

**Figure S2.** GO enrichment bubble plot of DEGs between JZ rootstock-grafted and HZ504 self-grafted tomato fruits.

**Figure S3**. Gene-metabolite association network of flavonoid biosynthesis in tomato fruits grafted onto JZ rootstock.

The network illustrates the significant correlations between differentially expressed genes (DEGs) and differentially accumulated metabolites (DEMs) involved in the flavonoid pathway. Red circles represent genes, blue triangles represent metabolites. Red lines indicate positive correlations, and green lines indicate negative correlations.
